# Supplementary figures and images for: CRISPR/Cas9 interrogation of the mouse Pcdhg gene cluster reveals a crucial isoform-specific role for Pcdhgc4
Source: PLoS Genet. 2019 Dec 26;15(12):e1008554. doi: 10.1371/journal.pgen.1008554 (PMC6957209; doi:10.1371/journal.pgen.1008554)

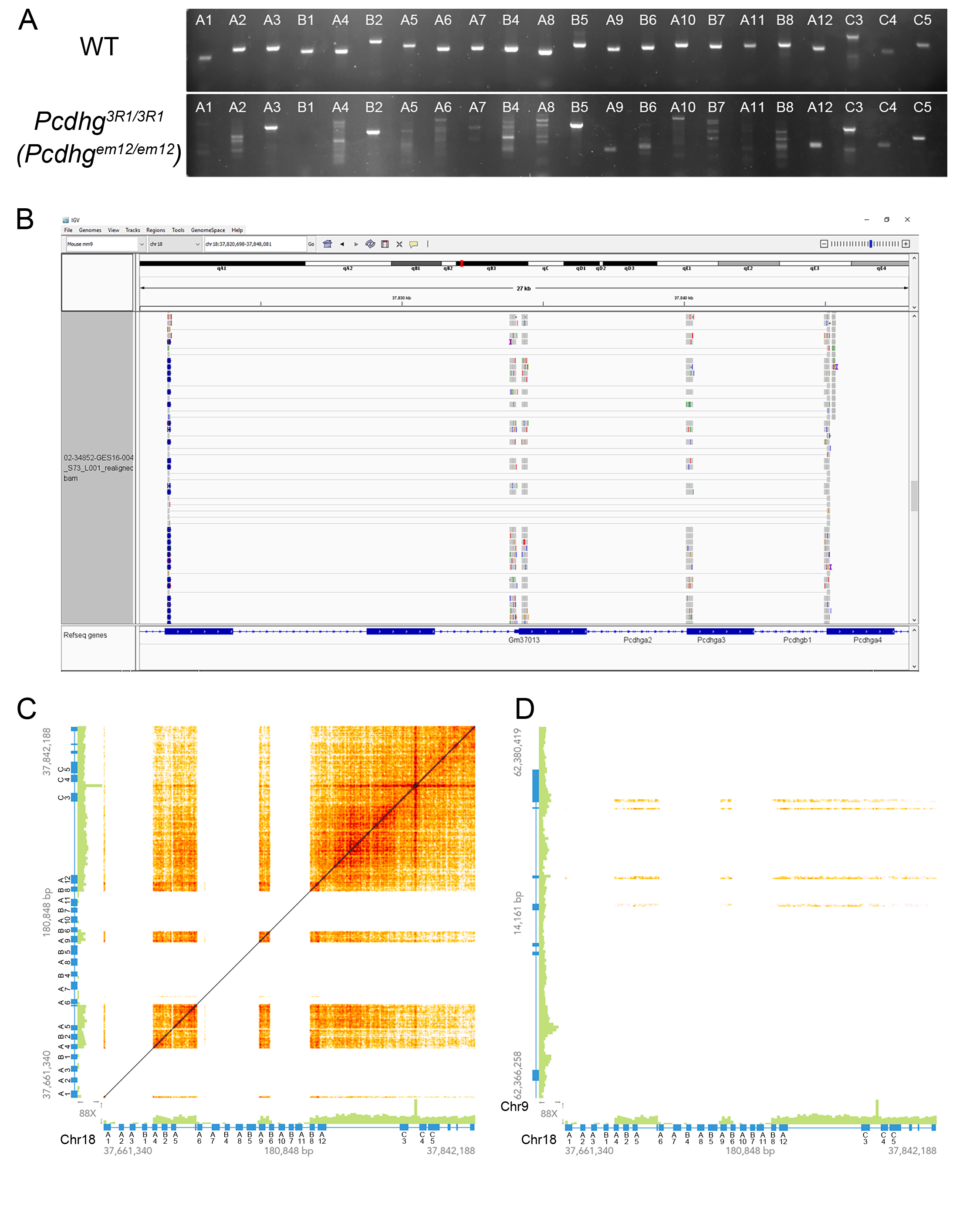

Supplement: S1 Fig — A) PCR from genomic DNA in 3R1 homozygous mutants compared with wild type, using primer pairs spanning each sgRNA target site. PCR products were either absent or abnormal in size from more exons than predicted by the custom amplicon sequencing analysis. B) Amplicon sequencing reads from a heterozygous 1R1 mutant were aligned to the mouse genome and visualized in the Integrated Genome Viewer (IGV) with paired-end reads highlighted. In this example, multiple read pairs map with one end in exon A1 and the other in exon A4, indicating a junction between A1 and A4 that was not detected by BreaKmer. C) Linked-read whole genome sequencing was performed on DNA from a homozygous 3R1 mutant. The matrix represents the locations of mapped reads with common barcodes, indicating that they originated from common DNA fragments within a microfluidic droplet. The Pcdhg locus is presented on the X and Y axes, revealing three large deletions including the deletion resulting in the A1-A4 fusion found in B. D). Linked-read sequencing also revealed that Anp32a coding sequence incorporated into the Pcdhg locus, as reads mapping to its exons (but not introns) had barcodes in common with those mapping to Pcdhg (Anp32a is on the Y axis, Pcdhg on the X axis). (TIF) [file pgen.1008554.s001.tif]

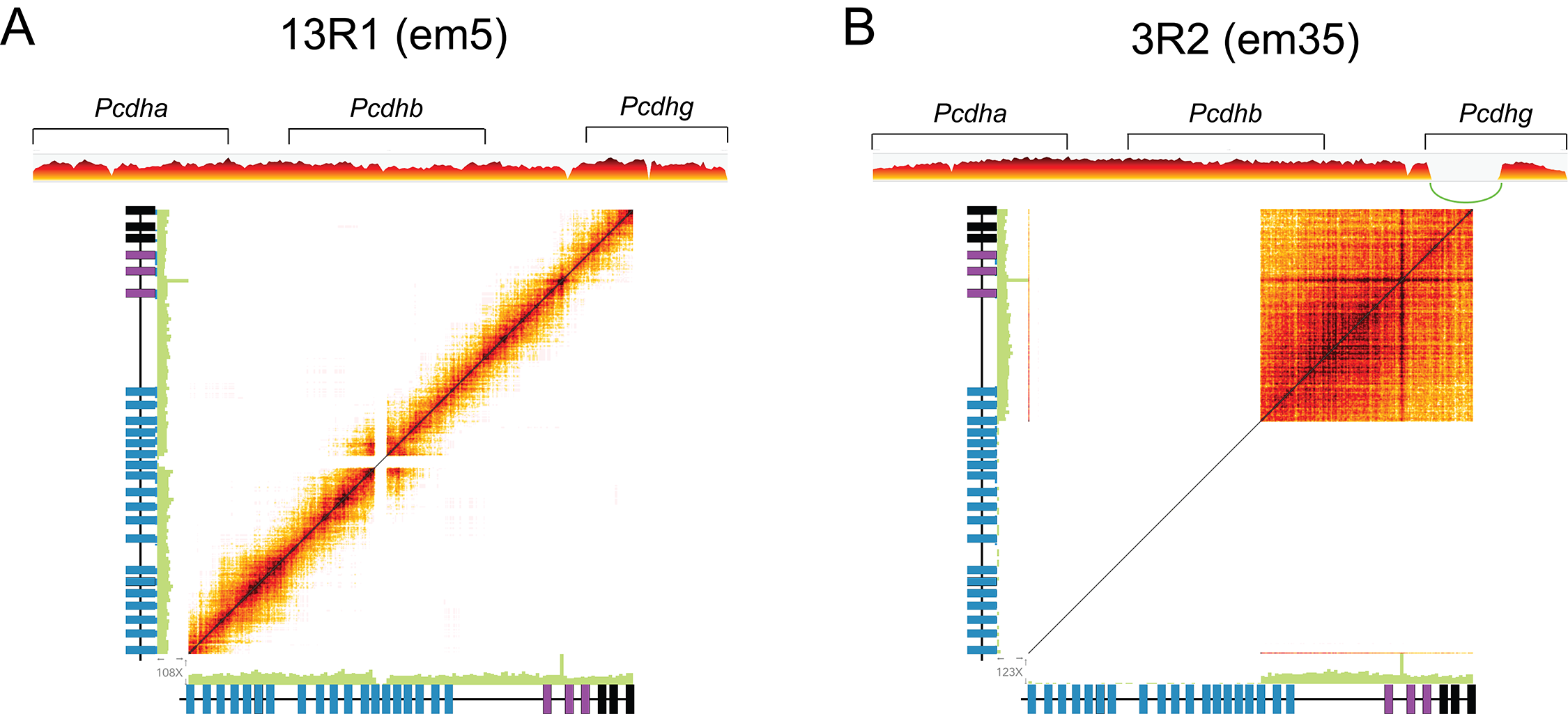

Supplement: S2 Fig — 10X Chromium linked-read sequencing results from (A) 13R1 and (B) 3R2 mutants demonstrate normal coverage through the Pcdha and Pcdhb clusters (upper panel), but coverage gaps where sequence was deleted in the Pcdhg locus (upper and lower panels). Short reads with the same barcode (i.e., from the same initial larger fragment) are connected on the matrix in the lower panel. Actual read sequences were used to identify junctions. (TIF) [file pgen.1008554.s002.tif]

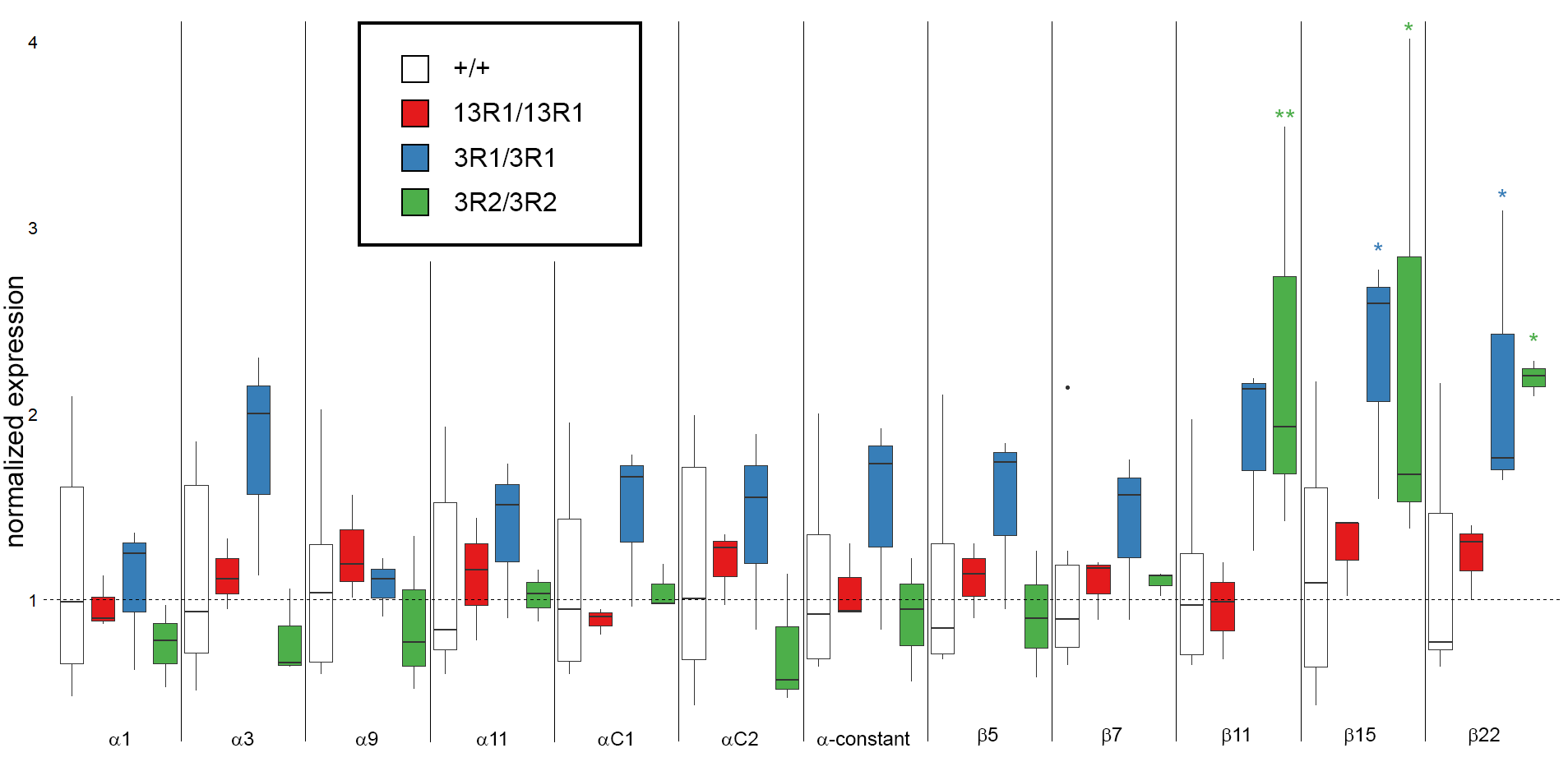

Supplement: S3 Fig — Quantitative real-time PCR of cDNA reverse-transcribed from RNA isolated form cerebral cortex from 13R1 mutants (red), 3R1 mutants (blue) and 3R2 mutants (green) demonstrated no change in isoform expression from the Pcdha cluster genes analyzed. Pcdhb isoforms at the 3’ end of the cluster were increased in mutants with large deletions in Pcdhg (i.e., in 3R1 and 3R2 mutants, but not in 13R1 mutants). * = p < 0.05; ** = p < 0.01 by Tukey post-hoc test comparing the indicated genotype with wild type. n = 3–9 animals per genotype. Box plots represent the median, first and third quartile, range, and outliers. (TIF) [file pgen.1008554.s003.tif]

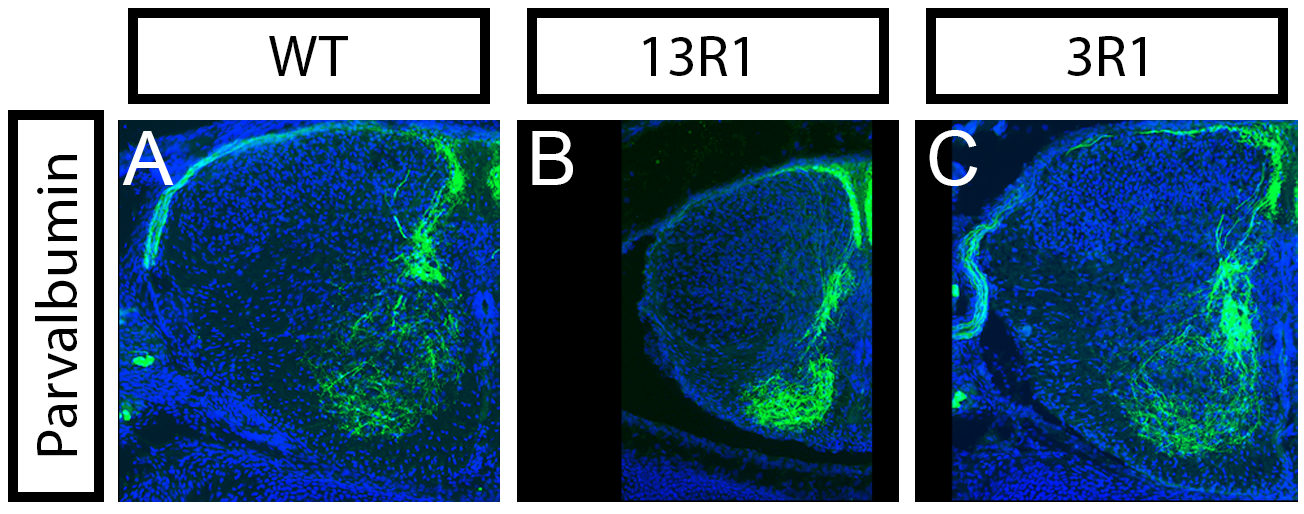

Supplement: S4 Fig — Parvalbumin staining of proprioceptive Ia afferent axons in spinal cord sections from P0 A) wild type, B) 13R1 homozygous mutants, and C) 3R1 homozygous mutants reveals axon terminal clumping in 13R1, but not 3R1 or wild type animals. (TIF) [file pgen.1008554.s004.tif]

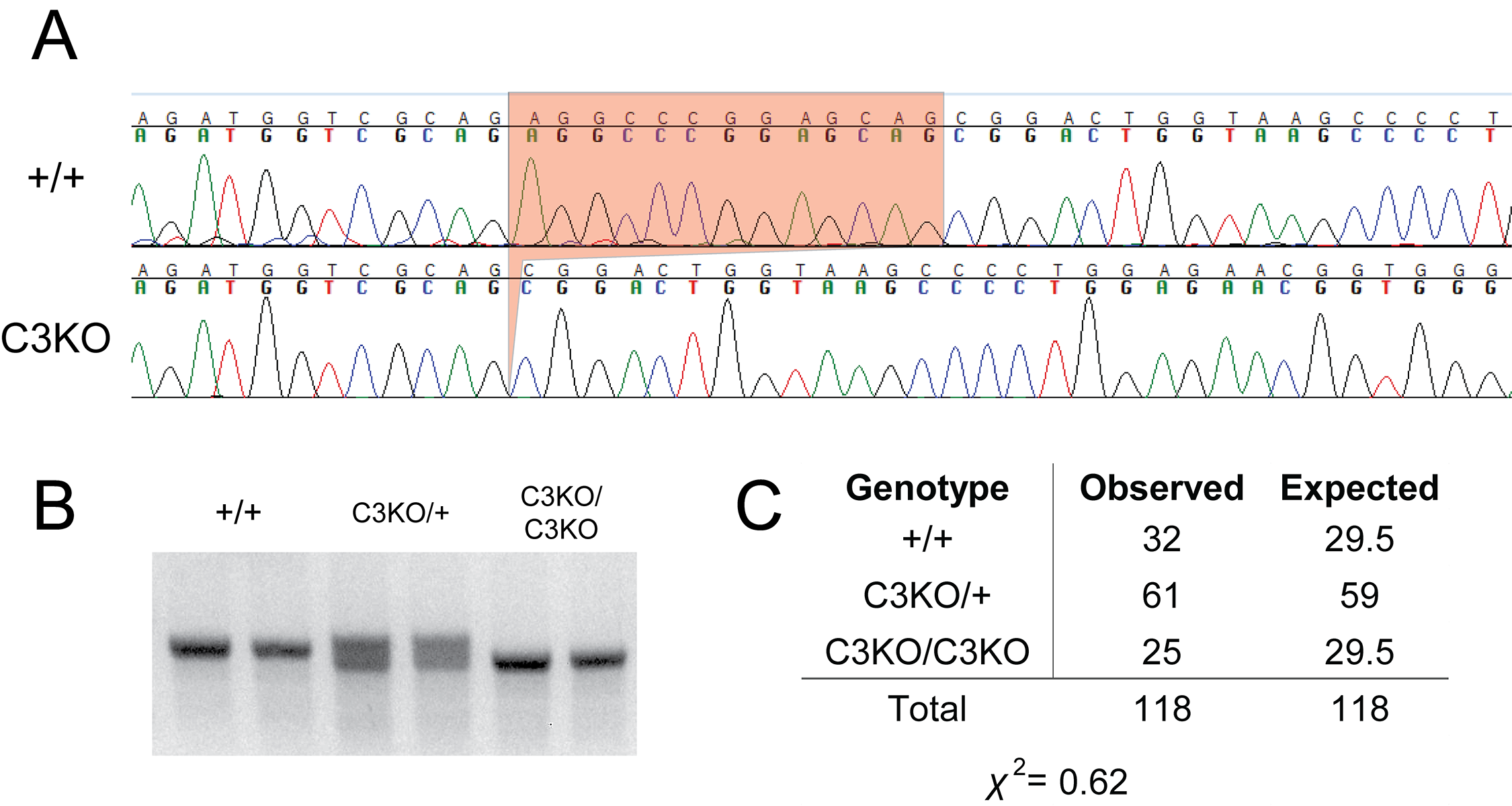

Supplement: S5 Fig — A) CRISPR/Cas9 targeting of Pcdhgc3 only resulted in a 13 bp deletion in the PcdhgC3KO allele (referred to as C3KO here). B) Tail genotyping PCR spanning the deletion was used to identify wild type, heterozygous, and homozygous mutants. C) Homozygous C3KO mutants survive in numbers not significantly different from the expected Mendelian ratio (n = 118 offspring from 18 litters). (TIF) [file pgen.1008554.s005.tif]

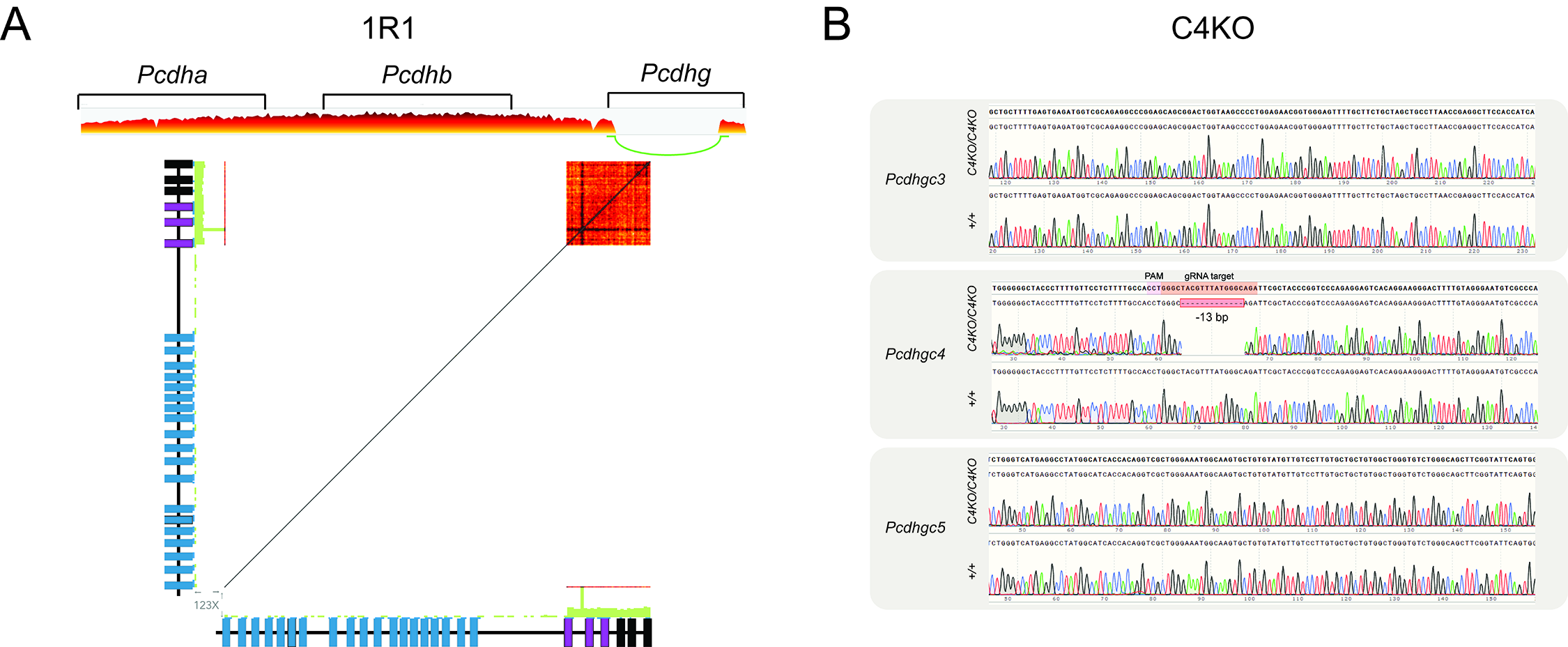

Supplement: S6 Fig — A) 10X Chromium linked-read sequencing results from 1R1 homozygous mutants demonstrates normal coverage through the Pcdha and Pcdhb clusters (upper panel), but a large gap between Pcdhga1 and Pcdhgc3 (upper and lower panels). Short reads with the same barcode (i.e., from the same initial larger fragment) are connected on the matrix in the lower panel. Actual read sequences were used to identify the junction. B) Sanger sequencing was performed on PCR from genomic DNA from C4KO homozygous mutants. A frame-shifting 13 bp deletion was identified at the guide site in Pcdhgc4, but no mutations were found in any of the other isoforms (Pcdhgc3 and Pcdhgc5 are shown here, all other isoforms were also sequenced). (TIF) [file pgen.1008554.s006.tif]

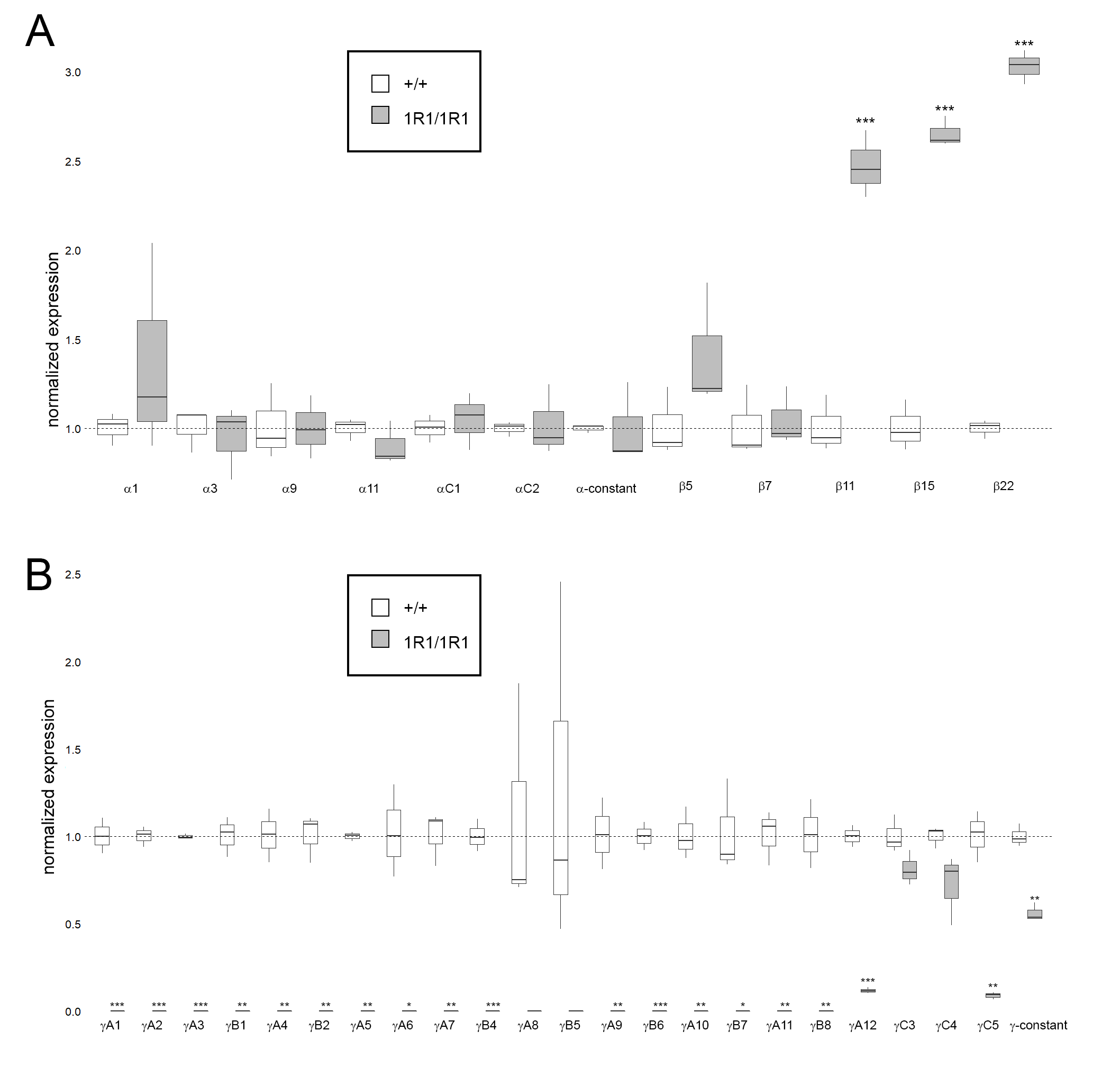

Supplement: S7 Fig — A) Quantitative real-time PCR of cDNA reverse-transcribed from RNA isolated form cerebral cortex from 1R1 mutants (gray) compared to control (white) demonstrated no change in isoform expression from the Pcdha cluster. Expression of Pcdhb isoforms at the 3’ end of the cluster was increased in 1R1 mutants, consistent with the effect from large deletions in 3R1 and 3R2 mutants. B) Expression of the Pcdhg cluster reflected genomic mutations, including expression from the γA1-γC3 fusion. γC5 isoform expression was significantly reduced (disrupted by 1 base pair insertion), and total γ-constant expression was reduced by half. * = p < 0.05; ** = p < 0.01; *** = p < 0.001 by student’s t-test. n = 3 animals per genotype. Box plots represent the median, first and third quartile, range, and outliers. (TIF) [file pgen.1008554.s007.tif]

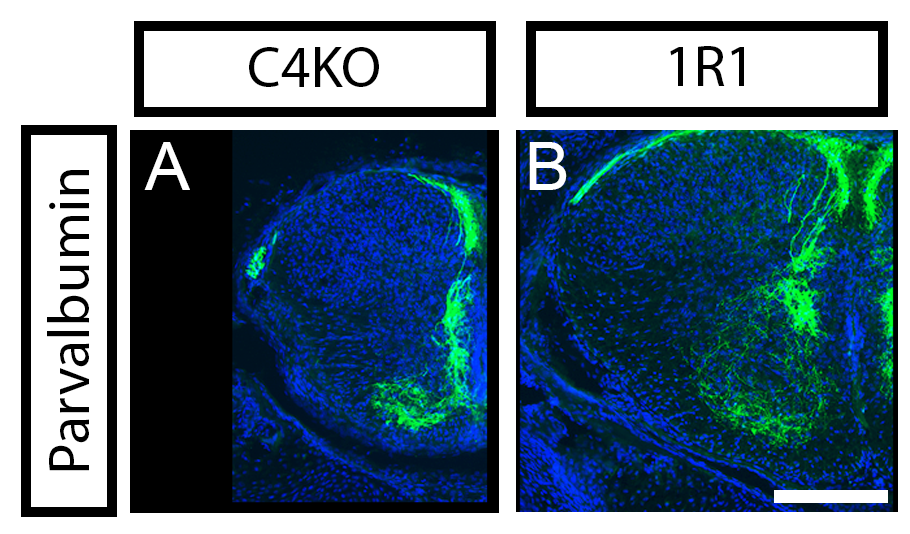

Supplement: S8 Fig — A) Parvalbumin staining in spinal cord sections from P0 C4KO mutants revealed aggregation of proprioceptive Ia afferent axons comparable to that observed in null animals or in 13R1. B) Conversely, 1R1 homozygous mutants exhibited normal terminal morphology similar to wild type, 3R1, and 3R2 pups. (TIF) [file pgen.1008554.s008.tif]
