# Supplementary material for: CRISPR/Cas9 interrogation of the mouse Pcdhg gene cluster reveals a crucial isoform-specific role for Pcdhgc4
Source: PLoS Genet. 2019 Dec 26;15(12):e1008554. doi: 10.1371/journal.pgen.1008554 (PMC6957209; doi:10.1371/journal.pgen.1008554)
Supplement: S1 Table — Mutations were identified by analyzing sequence results from the custom amplicon using GATK (blue = above threshold or purple = below threshold), Breakmer (orange), or visual inspection of aligned reads (yellow), or by analyzing whole genome sequencing (green). Numbers indicate the size of insertion (positive numbers) or deletion (negative numbers). Dashed lines indicate large scale rearrangements or deletions between the indicated exons. (PDF) [file pgen.1008554.s009.pdf]

F	sgRNA conc.	Allele	A1	A2	A3	B1	A4	B2	A5	A6	A7	B4	A8	B5	A9	B6	A10	B7	A11	B8	A12	C3	C4	C5
55-2	50 ng/μl	*Pcdhg^{em4}*			-2,-3	-5			-5,1,-1															
54-2	50 ng/μl	*Pcdhg^{em5}*	-2			-5			-34		2				[---	---	---]	-5					-5	
53-5	50 ng/μl	*Pcdhg^{em6}*	[---	---	------]				-6								-5	-6	-7	-9				
53-5	50 ng/μl	*Pcdhg^{em7}*			-4,-6												-70						-5	
52-4	50 ng/μl	*Pcdhg^{em8}*	[---	---	---	---	---	---	---	---	---	---	---	---	---	---	---	---	---	---	---	---]		1
52-4	50 ng/μl	*Pcdhg^{em9}*	[---	---	---	---	---	---	---]	3				2,7		-9					-10			
55-3	50 ng/μl	*Pcdhg^{em10}*							3								-70					-9	-9	-7
55-3	50 ng/μl	*Pcdhg^{em11}*	-12						-11															
54-3	50 ng/μl	*Pcdhg^{em12}*	[---	---	---	---	---]	52	[---	---	---	---	---	---	---	---	---	---	---	---]	---]			
43-4	10 ng/μl	*Pcdhg^{em13}*	-1	-76	-6,2									-1										
42-5	10 ng/μl	*Pcdhg^{em14}*	1		-2,-3				-3,-1,-6				-2,3,-1											
37-2	10 ng/μl	*Pcdhg^{em16}*												-19	-10							-2		
37-2	10 ng/μl	*Pcdhg^{em17}*	[---	---	---	---	---	---	---]							3					-10	-2		
37-2	10 ng/μl	*Pcdhg^{em19}*				[---	---	---	---	---	---	---	---	---	---	---	---	---	---	---]	-10			
54-3	50 ng/μl	*Pcdhg^{em23}*	[---	---	---	---	---	---	---	---	---]													
54-3	50 ng/μl	*Pcdhg^{em24}*																		-1				
54-1	50 ng/μl	*Pcdhg^{em25}*		-1	-1												-71		[---	---	---]			
55-1	50 ng/μl	*Pcdhg^{em27}*	[---	---	---	[---	---	---	---	---]	---	---]												
54-1	50 ng/μl	*Pcdhg^{em28}*	1		-7													-10						
55-3	50 ng/μl	*Pcdhg^{em31}*					3,-1														-10	-9	-9	-7
52-4	50 ng/μl	*Pcdhg^{em32}*			-3	[---	---	---	-6	---]	16													
43-4	10 ng/μl	*Pcdhg^{em33}*	1	-16	[---	---	---	---	------]			[---	---	---	---	---	---	---	---	---]	-10			
52-4	50 ng/μl	*Pcdhg^{em34}*	[---	---	---	---	---	---	------]					9		-9	-71							
55-3	50 ng/μl	*Pcdhg^{em35}*	[---	---	---	---	---	---	---	---	---	---	---	---	---	---	---	---	---]		-10		-9	-7
52-1	50 ng/μl	*Pcdhg^{em42}*	7											-6										
55-2	50 ng/μl	*Pcdhg^{em44}*																				-2		

N = Custom amplicon analysis (GATK, above threshold)
N = Custom amplicon analysis (GATK, below threshold)
N = Custom amplicon analysis (Breakmer)
N = Custom amplicon analysis (visual inspection of alignment)
N = Whole genome sequencing

S1 Table: Methods used to identify mutations.
