# Supplementary material for: CRISPR/Cas9 interrogation of the mouse Pcdhg gene cluster reveals a crucial isoform-specific role for Pcdhgc4
Source: PLoS Genet. 2019 Dec 26;15(12):e1008554. doi: 10.1371/journal.pgen.1008554 (PMC6957209; doi:10.1371/journal.pgen.1008554)
Supplement: S2 Table — (PDF) [file pgen.1008554.s010.pdf]

Name	sequence	note
Pcdha1QF	CCCAGGTTTGAACATAGC	Used with PcdhaQR
Pcdha3QF	GACAAACTGGTTGGAGACAT	Used with PcdhaQR
Pcdha9QF	TACACCTTGCCCAGTGG	Used with PcdhaQR
Pcdha11QF	CTGGGTAGAGATGAAAGGG	Used with PcdhaQR
PcdhaC1QF	GGGGATCATTCAAATGTGGA	Used with PcdhaQR
PcdhaC2QF	CAACAGGCAACTCACCG	Used with PcdhaQR
PcdhaQR	CGAGGCAGAGTAGCGCC	
PcdhaconQF	CGCTACTCTGCCTCGCTAA	
PcdhaconQR	GCTGTTGCTGTTGACACCG	
Pcdhb5QF	GGTGATAGAGGTGTTGGATGTG	
Pcdhb5QR	GATGATAGCGACCACAGTTTCT	
Pcdhb7QF	CTGGGAAAGGCTTGGTAATAGT	
Pcdhb7QR	GAGCTGGTCAGTGAAGATATGG	
Pcdhb11QF	AGCTGGATTTCGAGGGAATTAG	
Pcdhb11QR	CTCTGGAGCGTTGTCATTCA	
Pcdhb15QF	TGTCCCAGAACTACCAGTATGA	
Pcdhb15QR	GTGCTTTCGGTTAGGAAGTTAGA	
Pcdhb22QF	CTGGAACCTTCCGTTGAGAA	
Pcdhb22QR	GTCGGTGACGGAGATAGTAATG	
PcdhgA1QF	AGGAATTTTTGTCAGCACCCC	Used with PcdhgQR1
PcdhgA2QF	GATTTCCTCTCAGCACCTCAG	Used with PcdhgQR1
PcdhgA3QF	GCGAGCCTCTTATAATACCTCAAG	Used with PcdhgQR1
PcdhgA4QF	AGTGATCCTCTCCTGGTATCTC	Used with PcdhgQR1
PcdhgA5QF	ACACACAAAGAAGAGCCCG	Used with PcdhgQR2
PcdhgA6QF	TGCAAAGAGGAAGACTCTCTTGA	Used with PcdhgQR2
PcdhgA7QF	GAAGCCCCAAGTTCCCAG	Used with PcdhgQR2
PcdhgA8QF	GAATAAGGATGAAGATGCTTGCG	Used with PcdhgQR2
PcdhgA9QF	ACACTCCTTTGGTTCCTCAAG	Used with PcdhgQR2
PcdhgA10QF	TGATTCCAAGTGTCCTGTAGAAG	Used with PcdhgQR2
PcdhgA11QF	ACCGACAGATATTCAGCAAGC	Used with PcdhgQR2
PcdhgA12QF	CATGCATTAAATCAGCAAGCCC	Used with PcdhgQR2
PcdhgB1QF	TCCTTCTGTGGTTGTATGTGG	Used with PcdhgQR1
PcdhgB2QF	TGTCTCGGATTCTATCTCAAAGC	Used with PcdhgQR2
PcdhgB4QF	AATGAGTCAACCTCCCATCAG	Used with PcdhgQR2
PcdhgB5QF	TTCCCACCCCGAACCTCTAA	Used with PcdhgQR2
PcdhgB6QF	TTCCGGTAGTTCTCCTGGGG	Used with PcdhgQR1
PcdhgB7QF	TCCAGCCGCACAAGATATTC	Used with PcdhgQR1
PcdhgB8QF	CGAGACCTTTGTACGGAAGC	Used with PcdhgQR1
PcdhgC3QF	CTATAGACAGGTGTTGGGTGC	Used with PcdhgQR2
PcdhgC4QF	GTCCACCCTCTGATCTTCTCTA	Used with PcdhgQR2
PcdhgC5QF	CTTCACTTTCCTCAGGCCC	Used with PcdhgQR1
PcdhgQR1	TTGAGAGAAACGCCAGTCAG	
PcdhgQR2	GCATCTCTGTATCAAACTGGTTG	
PcdhgconQF	CCAACACTGACTGGCGTTTC	
PcdhgconQR	ATGGCTTGCAGCATCTCTGT	

S2 Table: Primers used for quantitative RT-PCR
