# Supplementary material for: CRISPR/Cas9 interrogation of the mouse Pcdhg gene cluster reveals a crucial isoform-specific role for Pcdhgc4
Source: PLoS Genet. 2019 Dec 26;15(12):e1008554. doi: 10.1371/journal.pgen.1008554 (PMC6957209; doi:10.1371/journal.pgen.1008554)
Supplement: S3 Table — (PDF) [file pgen.1008554.s011.pdf]

Allele name here	Strain name	Stock number
$Pcdhg^{em4}$	C57BL/6J-Pcdhg<em4Rwb>/Rwb	29164
$Pcdhg^{em5}$ ($Pcdhg^{13R1}$)	C57BL/6J-Pcdhg<em5Rwb>/Rwb	29165
$Pcdhg^{em6}$	C57BL/6J-Pcdhg<em6Rwb>/Rwb	29166
$Pcdhg^{em7}$	C57BL/6J-Pcdhg<em7Rwb>/Rwb	29167
$Pcdhg^{em8}$ ($Pcdhg^{1R1}$)	C57BL/6J-Pcdhg<em8Rwb>/Rwb	29168
$Pcdhg^{em9}$	C57BL/6J-Pcdhg<em9Rwb>/Rwb	29169
$Pcdhg^{em10}$	C57BL/6J-Pcdhg<em10Rwb>/Rwb	29170
$Pcdhg^{em11}$	C57BL/6J-Pcdhg<em11Rwb>/Rwb	29171
$Pcdhg^{em12}$ ($Pcdhg^{3R1}$)	C57BL/6J-Pcdhg<em12Rwb>/Rwb	29172
$Pcdhg^{em13}$	C57BL/6J-Pcdhg<em13Rwb>/Rwb	29173
$Pcdhg^{em14}$	C57BL/6J-Pcdhg<em14Rwb>/Rwb	29174
$Pcdhg^{em16}$	C57BL/6J-Pcdhg<em16Rwb>/Rwb	29176
$Pcdhg^{em17}$	C57BL/6J-Pcdhg<em17Rwb>/Rwb	29177
$Pcdhg^{em19}$	C57BL/6J-Pcdhg<em19Rwb>/Rwb	29179
$Pcdhg^{em23}$	C57BL/6J-Pcdhg<em23Rwb>/Rwb	29183
$Pcdhg^{em24}$	C57BL/6J-Pcdhg<em24Rwb>/Rwb	29184
$Pcdhg^{em25}$	C57BL/6J-Pcdhg<em25Rwb>/Rwb	29185
$Pcdhg^{em27}$	C57BL/6J-Pcdhg<em27Rwb>/Rwb	29187
$Pcdhg^{em28}$	C57BL/6J-Pcdhg<em28Rwb>/Rwb	29188
$Pcdhg^{em31}$	C57BL/6J-Pcdhg<em31Rwb>/Rwb	29191
$Pcdhg^{em32}$	C57BL/6J-Pcdhg<em32Rwb>/Rwb	29192
$Pcdhg^{em33}$	C57BL/6J-Pcdhg<em33Rwb>/Rwb	29193
$Pcdhg^{em34}$	C57BL/6J-Pcdhg<em34Rwb>/Rwb	29194
$Pcdhg^{em35}$ ($Pcdhg^{3R2}$)	C57BL/6J-Pcdhg<em35Rwb>/Rwb	29195
$Pcdhg^{em42}$	C57BL/6J-Pcdhg<em42Rwb>/Rwb	29161
$Pcdhg^{em44}$	C57BL/6J-Pcdhg<em44Rwb>/Rwb	29163
$Pcdhg^{em3}$ ($Pcdhg^{C3KO}$)	C57BL/6J-Pcdhg<em3Rwb>/Rwb	27939
$Pcdhg^{em41}$ ($Pcdhg^{C4KO}$)	C57BL/6J-Pcdhg<em41Rwb>/Rwb	33473

S3 Table: Summary of new alleles created here.
