# Supplementary material for: CRISPR/Cas9 interrogation of the mouse Pcdhg gene cluster reveals a crucial isoform-specific role for Pcdhgc4
Source: PLoS Genet. 2019 Dec 26;15(12):e1008554. doi: 10.1371/journal.pgen.1008554 (PMC6957209; doi:10.1371/journal.pgen.1008554)
Supplement: S4 Table — (PDF) [file pgen.1008554.s012.pdf]

exon	sgRNA	PAM
A1	GGGATCCGCATCGTTTCCAG	AGG
A2	GTGCCGGAGGAGATCGACAA	AGG
A3	TTTCAGGCAGATCCGCTACT	CGG
A4	CTCCTTACAAATCAGACCGC	AGG
A5	ACGCTGTGCGCGCCAGGAAA	GGG
A6	GCTCCGCTACTCTATTCCCG	AGG
A7	CATTCTCCTGGGGATGCGGT	GGG
A8	CCCCGCGAGCTGGCGGAGCG	CGG
A9	ACTGCTAGGGATGCTGTGGG	AGG
A10	CCCAGGGCATCCATAAGAAA	AGG
A11	ACAGCGGGATCAGCGCGCGG	CGG
A12	AACGGCGGGAGGACTGCAAA	GGG
B1	GCCCATCCACTACTCCATTC	CGG
B2	CGGTCCGGCCTGGTGGCTGC	AGG
B4/B5	GGCACTGCTGAGAGGGGCCG	GGG
B6/B7	GCGGACCGGCTGGCCCAGCG	CGG
B8	GCAAGTCCGGTACTCTATTC	CGG
C3	GGTAAGCCCCTGGAGAACGG	TGG
C4	TCTGCCCATAAACGTAGCCC	AGG
C5	TGGCATCACCACAGGTCGCT	GGG

S4 Table: sgRNA sequences
