# Supplementary material for: CRISPR/Cas9 interrogation of the mouse Pcdhg gene cluster reveals a crucial isoform-specific role for Pcdhgc4
Source: PLoS Genet. 2019 Dec 26;15(12):e1008554. doi: 10.1371/journal.pgen.1008554 (PMC6957209; doi:10.1371/journal.pgen.1008554)
Supplement: S5 Table — (PDF) [file pgen.1008554.s013.pdf]

Name	sequence
PCDHGA1KF	TCTCTGGAGCTACTGCTGGA
PCDHGA1KR	AGCTCTTCCCGGTCTATCCT
PCDHGA2KF	CCCCACTGTGCTGAAAGCTA
PCDHGA2KR	CCTCCCTGTCTATCCTGCCT
PCDHGA3KF	CCATCGTGGGAATCAGAGGG
PCDHGA3KR	ACACAGCTCTTCCCGGTCTA
PCDHGA4KF	CACCAGCGTCCAACAGTACA
PCDHGA4KR	GATTACCCACCACAGAGCCC
PCDHGA5KF	TCTTGGGGAAAGCCACCATC
PCDHGA5KR	CTGGAGATAATGCGGACCCC
PCDHGA6KF	TGGATTACCAAGTGGGCTGG
PCDHGA6KR	TGGAGATGATGCGAATCCCG
PCDHGA7KF	CGGACACAACTGGGTTCTGA
PCDHGA7KR	ACCTTTGGTGATGATGCGGA
PCDHGA8KF	CAGCGTAGGACAGATTCGCT
PCDHGA8KR	CAAGACAAGGCGTGCTCTGA
PCDHGA9KF	GCTCCGGGACTACAGCAAAA
PCDHGA9KR	GGCACGGAATAGCGGATTTG
PCDHGA10KF	CTTTAAGGTGCTTCCGCTGC
PCDHGA10KR	CCTCTGGAGATGATGCGGAC
PCDHGA11KF	TTTGGATGCGGGATACCTGG
PCDHGA11KR	CCTGGAGATGATGCGGACTC
PCDHGA12KF	CCCTCTAGGAGCAAACTGGC
PCDHGA12KR	AGCCCCAGATCCTTGGAGAT
PCDHGB1KF	TACCTGGCAAGCTCAGAACG
PCDHGB1KR	CCCCACTCTCTGCACTAACG
PCDHGB2KF	AGACGCGTTAGGAAACTGGG
PCDHGB2KR	AATCCTTCTCTGCGCTGACC
PCDHGB4KF	GAGCCCGGAATATCCACACC
PCDHGB4KR	CTTTCGCGTCGGTAACTCCT
PCDHGB5KF	GCAAGGAAAGGGGAGCAAGA
PCDHGB5KR	CACCAGCTTCTTCCCGCATA
PCDHGB6KF	CCTCGTACCCTGCTGTGAAG
PCDHGB6KR	CTCCGAATCCACGCTGAAGT
PCDHGB7KF	ACCCTCGTAGGCTGAACTCT
PCDHGB7KR	CTCCGAATCCACGCTGAAGT
PCDHGB8KF	GAAGCCTCCTCCCTTCACAC
PCDHGB8KR	CAGAGGGCTTTGGAAGCAGA
PCDHGC3KF	CCGGGATGAGGCAGAGACTGAA
PCDHGC3KR	TTACAGTGCAGGAGGGCAGCGT
PCDHGC4KF	AGAATTAGCGGATGGCAGCA
PCDHGC4KR	TTTGGTTCACCTCTCCAGCG
PCDHGC5KF	TTCAGCTTCTGCACTCCAGG
PCDHGC5KR	CAAAGCTCCGCTCACCAAAC

S5 Table: Primers used to amplify target regions of *Pcdhg* variable exons.
